# *NCDN* is a Potential Biomarker and Therapeutic Target for Glioblastoma

**DOI:** 10.7150/jca.90535

**Published:** 2024-01-01

**Authors:** Xiaokai Huang, Chengwu Xu, Haipeng Dai, Jianchun Yang, Tingting Huang, Shuan Chen, Lingxin Qi, Jichen Ruan, Juxiang Wang

**Affiliations:** 1 Department of Hematology, The Second Affiliated Hospital and Yuying Children's Hospital of Wenzhou Medical University, Wenzhou 325027, Zhejiang, China.; 2 The Key Laboratory of Pediatric Hematology and oncology Diseases of Wenzhou, the Second Affiliated Hospital and Yuying Children's Hospital of Wenzhou Medical University, Wenzhou 325027, Zhejiang, China.; 3 Department of Pediatric Surgery, The Second Affiliated Hospital and Yuying Children's Hospital of Wenzhou Medical University, Wenzhou 325027, Zhejiang, China.

**Keywords:** glioblastoma, *NCDN*, TCGA data analysis, migration, apoptosis

## Abstract

**Background:** Glioblastoma (GBM) is a type of central nervous system malignancy. In our study, we determined the effect of *NCDN* in GBM patients through The Cancer Genome Atlas (TCGA) data analysis, and studied the effects of *NCDN* on GBM cell function to estimate its potential as a therapeutic target.

**Methods:** Gene expression profiles of glioblastoma cohort were acquired from TCGA database and analyzed to look for central genes that may serve as GBM therapeutic targets. Then the cell function of *NCDN* in glioblastoma cell was explored through in vitro cell experiments.

**Results:** Through gene ontology (GO) analysis, weighted gene co-expression network analysis (WGCNA), and survival analysis, we identified three key genes (*NCDN*, *PAK1* and *SPRYD3*) associated with poor prognosis in glioblastoma. In vitro experiments showed impaired cell migration, apoptosis, and cell cycle arrest in *NCDN* knockdown cells.

**Conclusion:** *NCDN* affects the progress and prognosis of glioblastoma by promoting cell migration and inhibiting apoptosis.

## Background

Glioblastoma is the most aggressive type of intracranial malignancy, accounting for 33% of all intracranial tumors 1. It can be diagnosed by a variety of techniques such as positron emission tomography, computed tomography, and magnetic resonance imaging 2. At present, the treatment method of GBM is mainly surgery, supplemented by chemoradiotherapy with the standards chemotherapy drug temozolomide 3, 4. However, due to the heterogeneity and complexity of tumor cells, high local aggressiveness and prominent neovascularization, patients with GBM have a poor prognosis 5, 6. In addition, glioblastoma harbors stem cells, which have the ability of differentiation and self-renewal and are highly resistant to radiotherapy and chemotherapy, which is further affecting the prognosis and survival rate of patients 7, 8. Hence, it is necessary to explore new therapeutic methods in clinical treatments of GBM.

In order to improve the prognosis for patients with GBM, many teams have tried to find new treatments, including monoclonal antibodies, small molecule inhibitors and cancer vaccines 9, 10. Meanwhile, emerging tumor inhibitors targeting DNA damage/repair pathways, tumor suppressor protein p53, growth factor receptors, cell cycle control enzymes/genes, and their downstream pathways, are used as alternative/supplementary anti-cancer strategies. These targeted drugs can also be used as radiosensitizers to enhance the cytotoxicity of radiation therapy while minimizing harmful side effects on surrounding normal tissue 11. Therefore, we aim to expand the list of molecular targets and to design novel small molecule inhibitors with central nervous system penetration to gain new insights for GBM treatments.

NCDN, a 79 kDa-sized cellular protein, is highly conserved in vertebrates 12. Its expression in nerve cells is relatively specific, while its expression in skeletal muscle, heart, kidney, and myeloid cells is much less 13-16. In nerve cells, NCDN is localized to the cyton, dendritic axis, and dendritic spines in a vesicle-like structure, and is able to maintain cell polarity by regulating dendritic morphogenesis and nerve cell signaling pathways 17-19. It has been found that the absence of *NCDN* can increase the production of reactive oxygen species, thus affecting a wide range of pathogens and signaling pathways. It also plays a role in intracellular transport by regulating the localization of signal proteins such as P-Rex1 20, 21. As an endogenous regulator of mGluR5, NCDN can negatively regulate the phosphorylation of Ca2+/calmodulin-dependent protein kinase II, affect neurite growth and synaptic plasticity 22. In addition, anti-NCDN is found to be a novel antibody related to autoimmune ataxia, therefore, NCDN can be used as a potential target antigen for autoimmune neurodegenerative diseases 23, 24. However, we did not find any studies on the effect of *NCDN* on GBM, and the clinical application value of *NCDN* remains to be clarified.

In our study, the RNA sequencing data of GBM samples were acquired from TCGA to select key genes associated with glioblastoma prognosis. Three genes (*NCDN*, *PAK1*, and *SPRYD3*) associated with the prognosis of GBM were selected by GO analysis, functional enrichment, WGCNA and survival analysis. In vitro cell models were subsequently established to investigate the effects of these selected key genes on the cellular functions in glioblastoma cell lines.

## Materials and methods

### Data sources and processing

Clinical information and RNA sequencing data of patients were downloaded from the 'GBM' cohort of TCGA (https://www.cancer.gov/about-nci/organization/ccg/research/structural-genomics/tcga) (**Table 1**). After excluding the samples that had undergone chemotherapy and radiotherapy before, six normal samples and 794 GBM samples were selected for follow-up analysis.

### Acquisition of GBM differentially expressed genes (DEGs)

Limma differential analysis was performed using the R software package limma (version 3.40.6) to select DEGs between case and control groups. Specifically, lmFit function was used for multiple linear regression, and eBays function was further used for Computed T-statistics, Moded F-statistics, and log-odds of differential expression. We considered *P* < 0.05 to be statistically significant.

### Functional enrichment of DEGs

Enrichment analysis was performed by R package clusterProfiler (version 3.14.3). We considered *P* < 0.05 to be statistically significant.

### WGCNA and acquisition of GBM hub genes

Gene expression profiling was employed to remove genes with a standard deviation of 0 in each sample, the R package WGCNA's goodSamplesGenes method was used to remove outliers and samples. WGCNA was further used to build scale-free co-expression networks. We calculated the corresponding module members (MM) and gene significance (GS) of each gene, and identified hub genes based on the cut-off criteria (|GS| > 0.6 and |MM| > 0.8).

### Survival analysis of hub genes

We analyzed the survival by R packet survival, and evaluated the prognostic significance of each gene by Cox regression analysis.

### Glioma tissue samples

Human GBM cell lines (U-251 MG, U-87 MG) and human astrocytes (NHA) were obtained from Beijing BNA Chuanglian Biotechnology Institute. Short tandem repeat analysis showed correct cell typing results.

### Quantitative reverse transcription PCR (RT-qPCR) analysis

Cells were lysed with TRIzol Reagent from Solarbio (Ribobio, Guangzhou, China), total RNA was extracted, and the RNA concentrations were determined by micronucleic acid detector (Zheke Instrument Equipment Co., Ltd, Zhejiang, China). cDNA was obtained using a reverse transcription kit (Vazyme, Nanjing, China) and amplified via fluorescence quantitative PCR (Bio-Rad, Zhejiang, China). Relative expression levels were calculated with the 2^-△△Ct^ method using *ACTB* as the reference gene. The primer sequences of *NCDN* used for PCR are as follows (from 5' to 3'):

Forward: CTGCCTGACAGGGTGGAGATTG

Reverse: TGGGACTGTGATAGAGAGGATGG

### Cell transfection

For transfecting the cells with the plasmid of *NCDN* shRNA (Guangzhou, China), LipofectamineTM3000 Reagent (ThermoFisher, Shanghai, China) was utilized. The cells were inoculated in 6-well plates and cultured to a density of 30% at 37 °C. Following replacement with Opti-MEM medium, a mixture of plasmid and LipofectamineTM3000 was added. The cells were further cultivated for 48 hours and the follow-up experiment was conducted. Target sequence of *NCDN* shRNA plasmid: CAAAGCAGGTGACATAGAT.

### Transwell assay

After transfection in six-well plates, cell densities were adjusted to 5 × 10^4^/100 µL. Complete medium (600 µL) containing 20% fetal bovine serum was added to a 24-well plate and a transwell chamber was placed on top. 200 µL of the cell suspension was added to the transwell chamber, incubated in 37 °C for 48 hours. Cells were subsequently fixed with 4% paraformaldehyde, and stained with 0.1% crystal violet. Images were obtained under a microscope and subjected to image-based quantification analyses and statistical analyses.

### Cell cycle analysis

2 × 10^6^ cells were collected and 5 mL 70% ethanol was added. Then the cells were incubated at 4 °C for 4 hours to be fixed and the washed twice with PBS. The staining buffer was mixed with 500ul PI/RNase and incubated with the fixed cells at 37 °C for 30 min. Stained cells were then analyzed by flow cytometry.

### Apoptosis Assay

After being counted, the cells to be tested were washed with PBS, centrifuged at 1000 rpm for five min. The supernatant was discarded, and the cells were resuspended with 1 × binding buffer. 100 ul cell suspension was transferred into the flow tube, 5 ul Annexin V staining solution and 10ul PI staining solution (Beyotime Biotechnology, Shanghai, China) were added. Fluorescence detection and data analysis were performed on a flow cytometer.

### Statistical analysis

Data analysis and mapping were performed using GraphPad Prism 8. Chi-square and T tests were employed to compare differences between experimental and control groups. We considered *P* < 0.05 to be statistically significant.

## Results

### Data pre-processing

**Figure 1:**
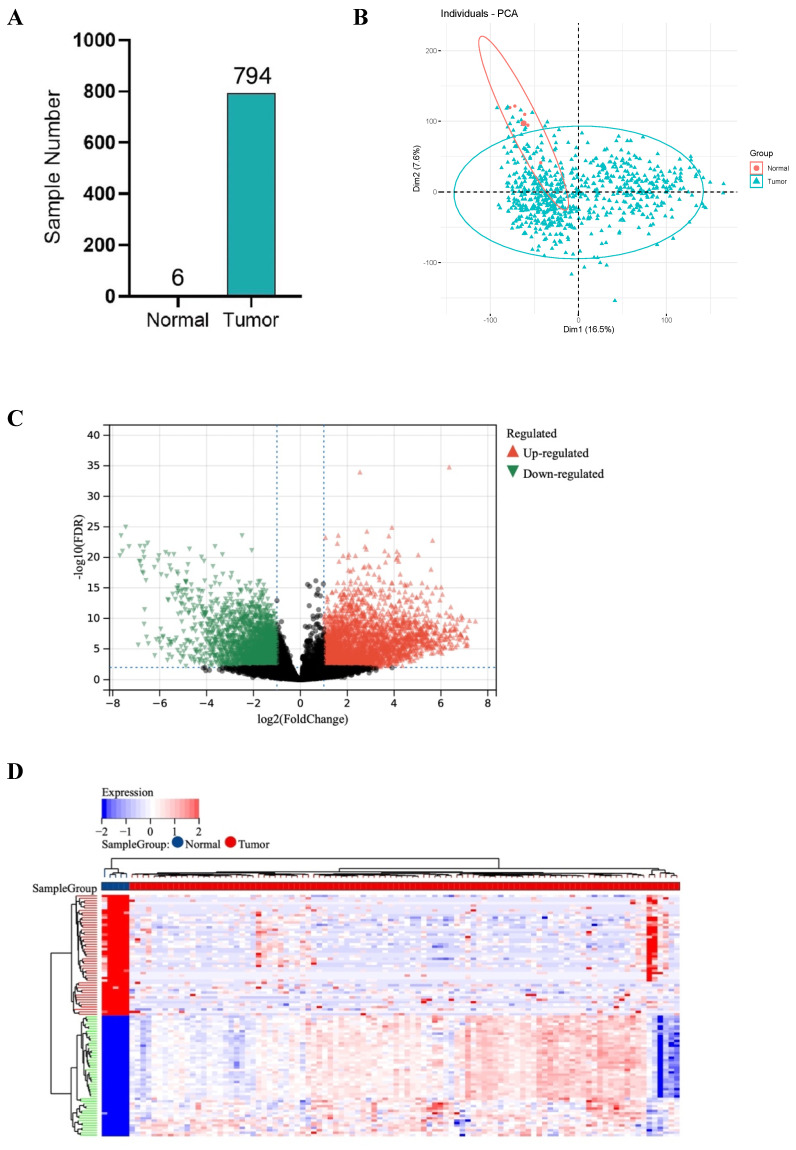

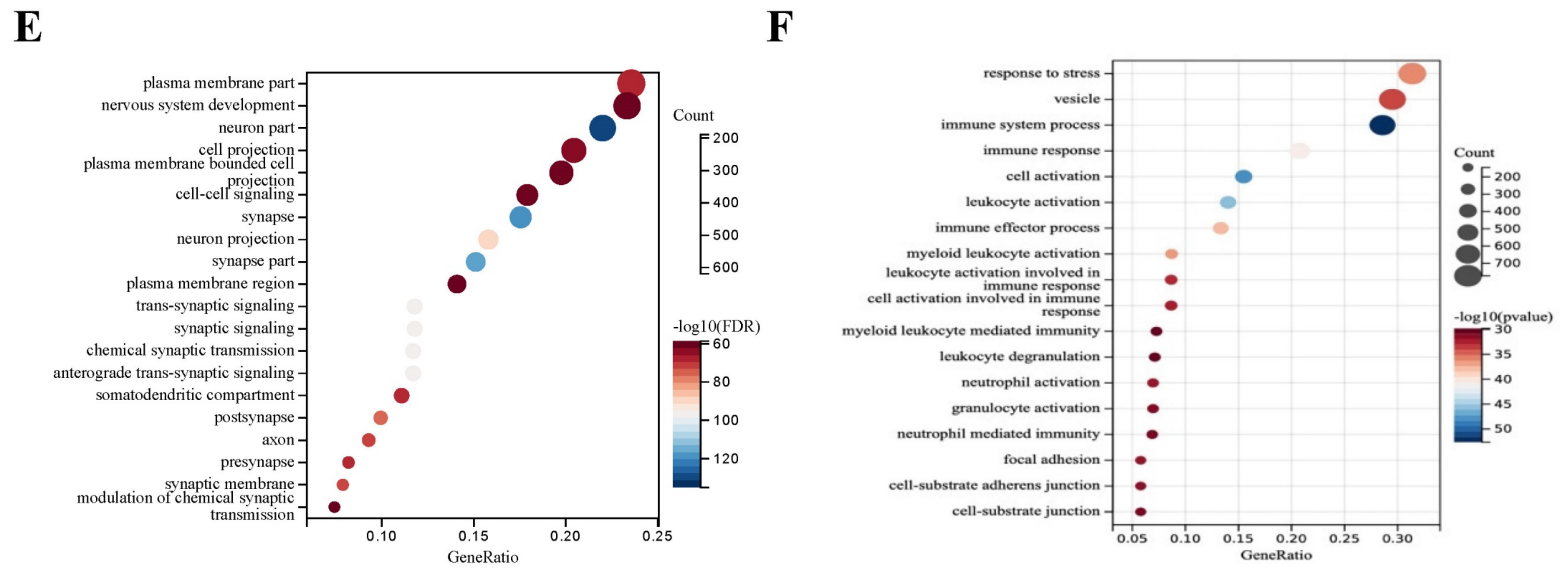
DEGs between 794 GBM and 6 normal samples. (A) Number of samples in case group and control group. (B) Principal component analysis. (C) The volcano plot of gene expression in normal and GBM samples: the red, green and black dots represent up-regulated, down-regulated and non-significantly changed genes in the GBM samples, respectively. (D) Heat map: blue dots, red dots, and gray dots represent down-regulated, up-regulated, and undifferentiated genes in GBM, respectively. (E) GO enrichment analysis of GBM up-regulated genes. (F) GO enrichment analysis of GBM down-regulated genes.

RNA sequencing data of GBM samples from TCGA for a total of 1132 cases were initially acquired and after excluding the patients who received chemotherapy and radiotherapy before, 6 normal samples and 794 tumor samples were finally included in this study. Tumors were successfully distinguished from normal samples by principal components, accounting for 16.5% and 7.6% of the observed differences (**Figure 1A, B**).

### Acquisition of DEGs and GO enrichment analysis

1527 up-regulated and 1986 down-regulated genes were identified (**Figure 1C, D**). The GO enrichment analysis results showed that up-regulated DEGs are mainly involved in neurological development, ion transmembrane transport (**Figure 1E**). In contrast, down-regulated DEGs are mainly involved in immune response and cell activation (**Figure 1F**). These results are consistent with the current findings of GBM dysfunction, suggesting that these results are credible for further analyses.

### WGCNA

**Figure 2:**
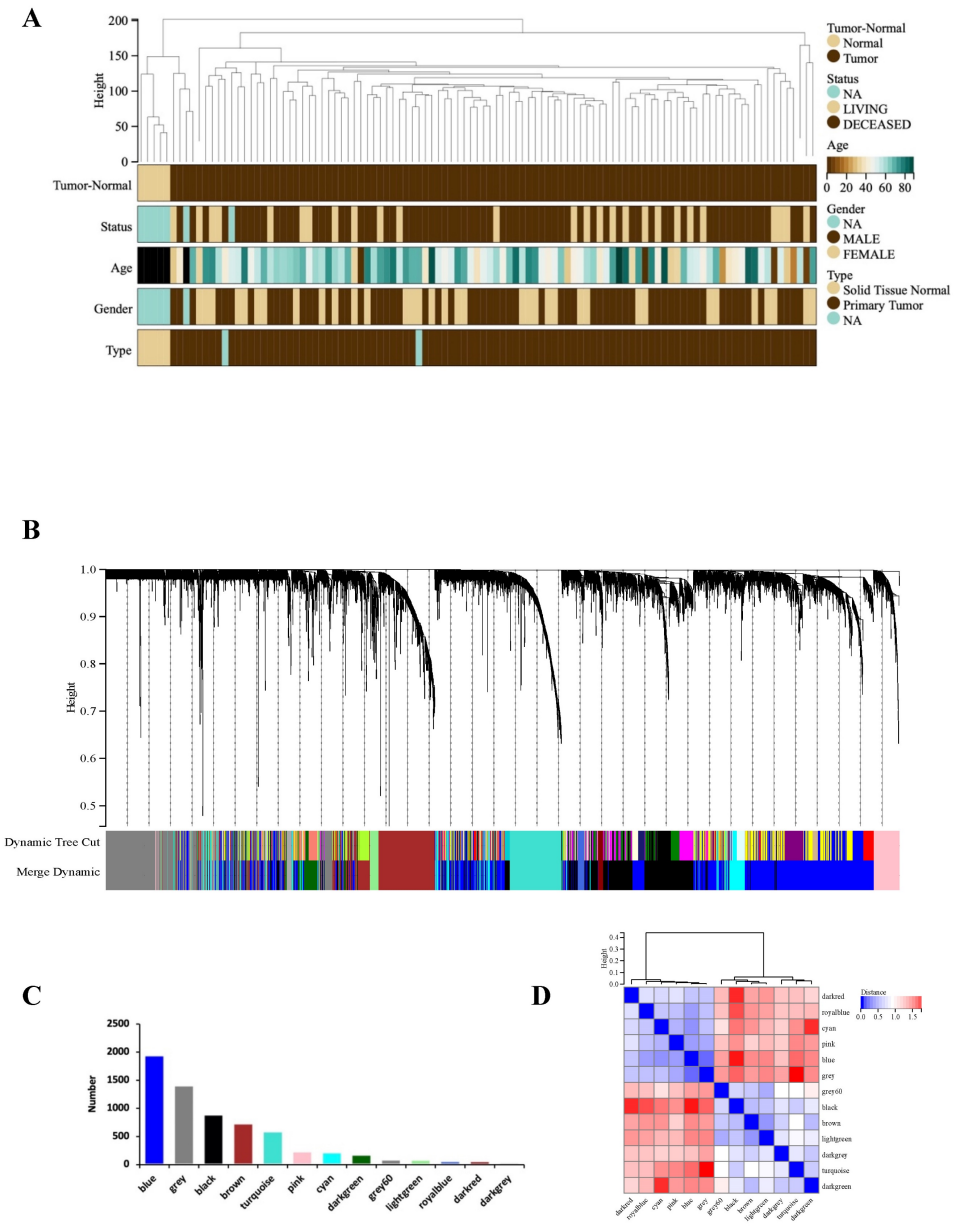

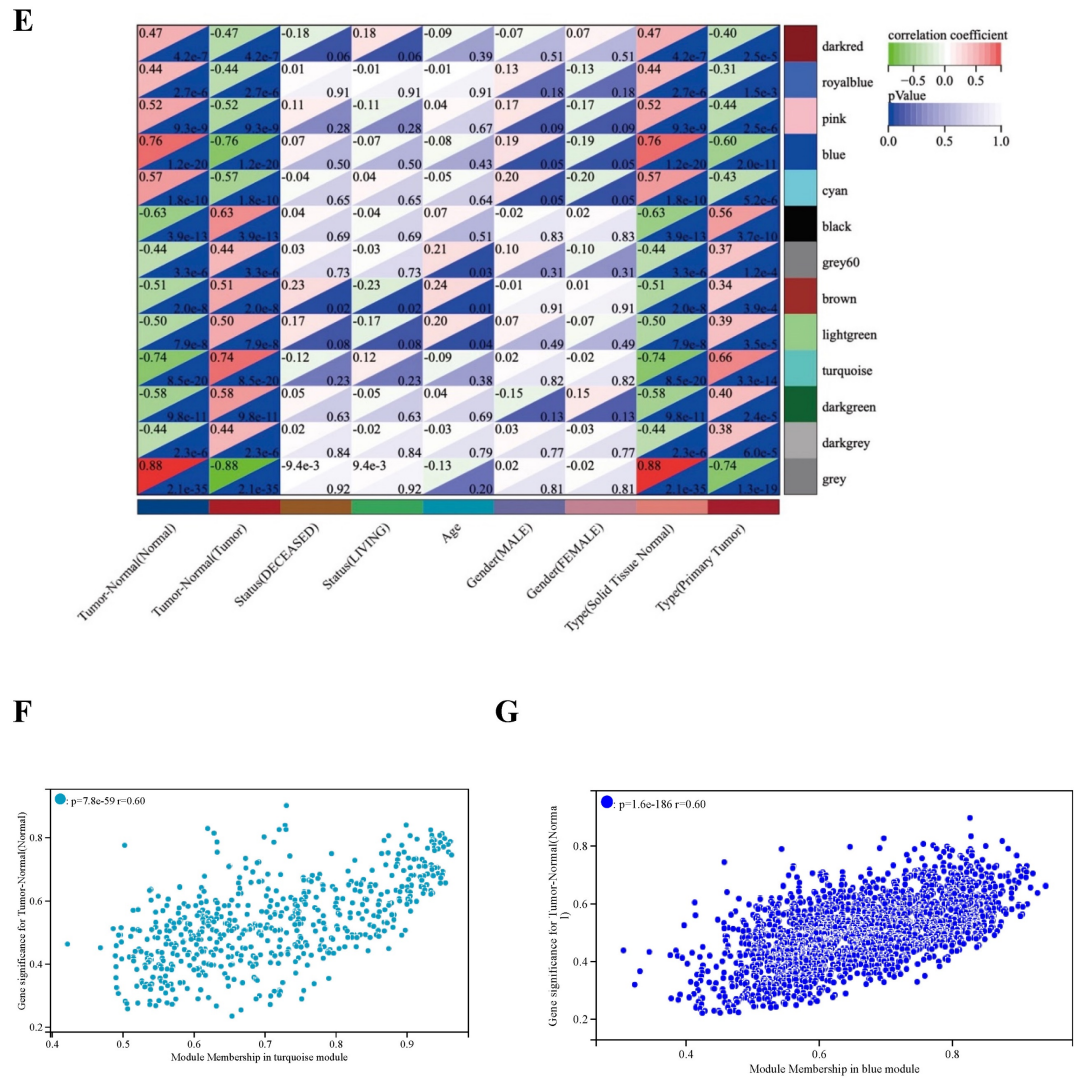
WGCNA analysis. (A) Clustering dendrogram: Brown color represents 'tumor' for the variable 'Tumor Normal', 'deceased' for the variable 'Status', 'low onset age' for the variable 'Age', and 'male' for the variable 'Gender', 'primary tumor' for the variable 'Type'. (B) Gene clustering: each color represents a module, and each branch represents a gene. (C) The number of genes in each module. (D) Module heatmap of eigengene adjacency. (E) Correlation between modular characteristic genes and clinical characteristics in patients with GBM. (F) (G) Scatter plots of GS score and MM for genes in turquoise module and blue module.

WGCNA analysis was performed based on the expression matrix of the 3513 DEGs and clinical data of 106 GBM samples. Firstly, 106 samples were clustered, all of which were clustered and within the critical threshold (height < 200) with no outliers removed (**Figure 2A**). WGCNA applied 5 clinical variables (**Figure 2A**): tumor-normal, status, age, sex, type.

According to the gene expression pattern, the obtained differential genes were grouped into different modules. The process yielded 13 co-expression modules: blue, black, brown, turquoise, pink, cyan, darkgreen, grey, lightgreen, royalblue, darkred, darkgrey, grey (**Figure 2B, C**).

The characteristic genes of blue module were negatively correlated with GBM (cor = -0.76, *P* = 1.2×10^-20^), while the characteristic genes of turquoise module were positively correlated with GBM (cor = 0.74, *P* = 0.74×10^-20^) (**Figure 2D**). The correlations are further confirmed by heat map (**Figure 2E**). Hence, the blue and turquoise modules were analyzed to reveal hub genes.

### Acquisition of candidate hub genes from blue and turquoise modules

The results show that there is a significant positive correlation between MM and GS scores in turquoise and blue modules (**Figure 2F, G**). In the turquoise and blue modules, we identified 588 genes and 1941 genes were identified to meet the thresholds of 'cor. gene MM' > 0.8 and the 'cor. gene GS' > 0.6, respectively.

### Survival analysis of hub genes

**Figure 3:**
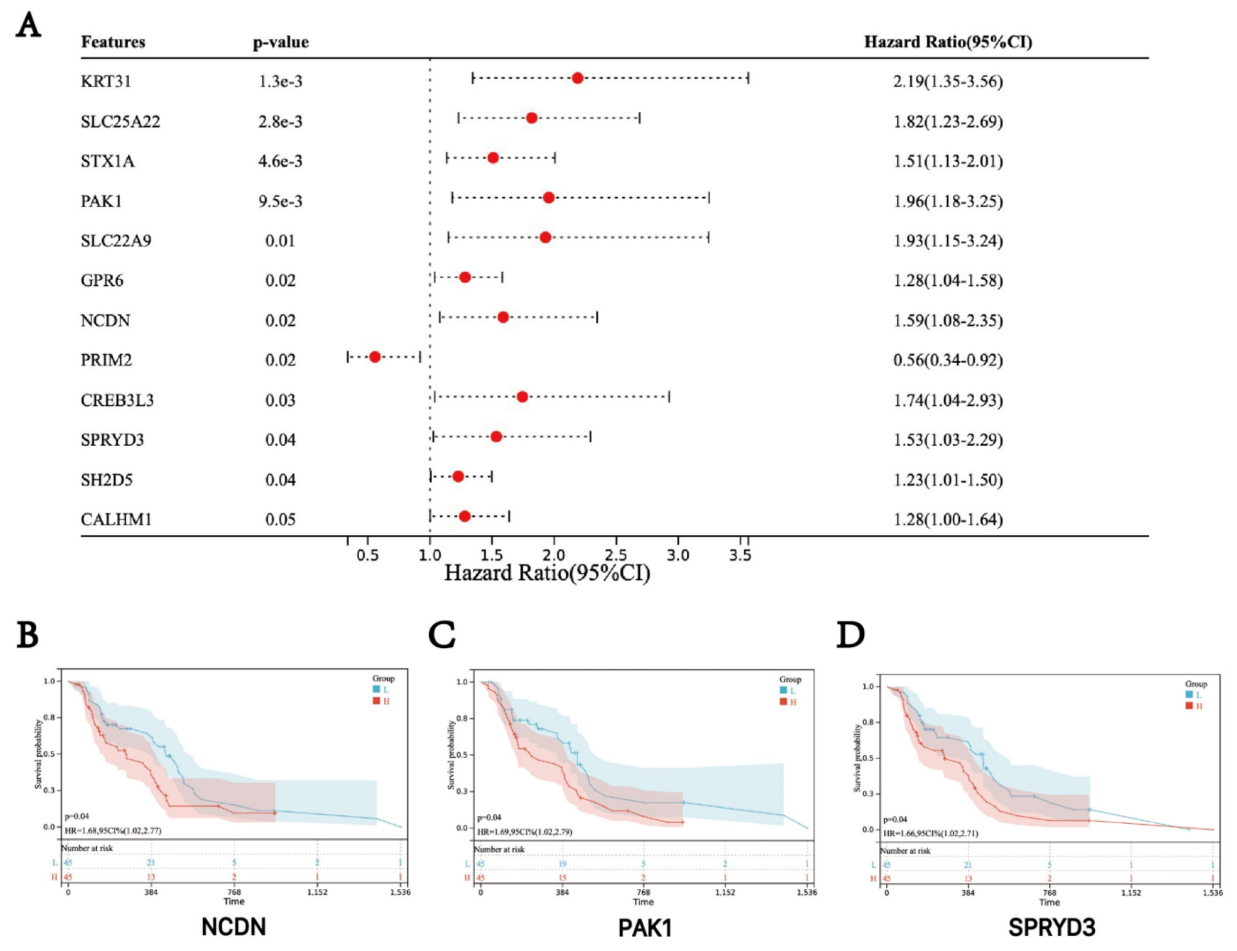
Survival analysis of three hub genes. (A) Forest plot of the hub genes. (B-D) Kaplan-Meier survival curves of GBM patients.

Based on the clinical information and expression data of the 106 GBM tumor samples, we examined the potential association between hub genes expression and patient survival. The analysis showed that genes in the turquoise module, including *NCDN*,* PAK1* and *SPRYD3* are associated with the prognosis of GBM. Therefore, these genes were defined as "final" central genes (**Figure 3**).

### The *NCDN* gene is upregulated in glioblastoma cells

**Figure 4:**
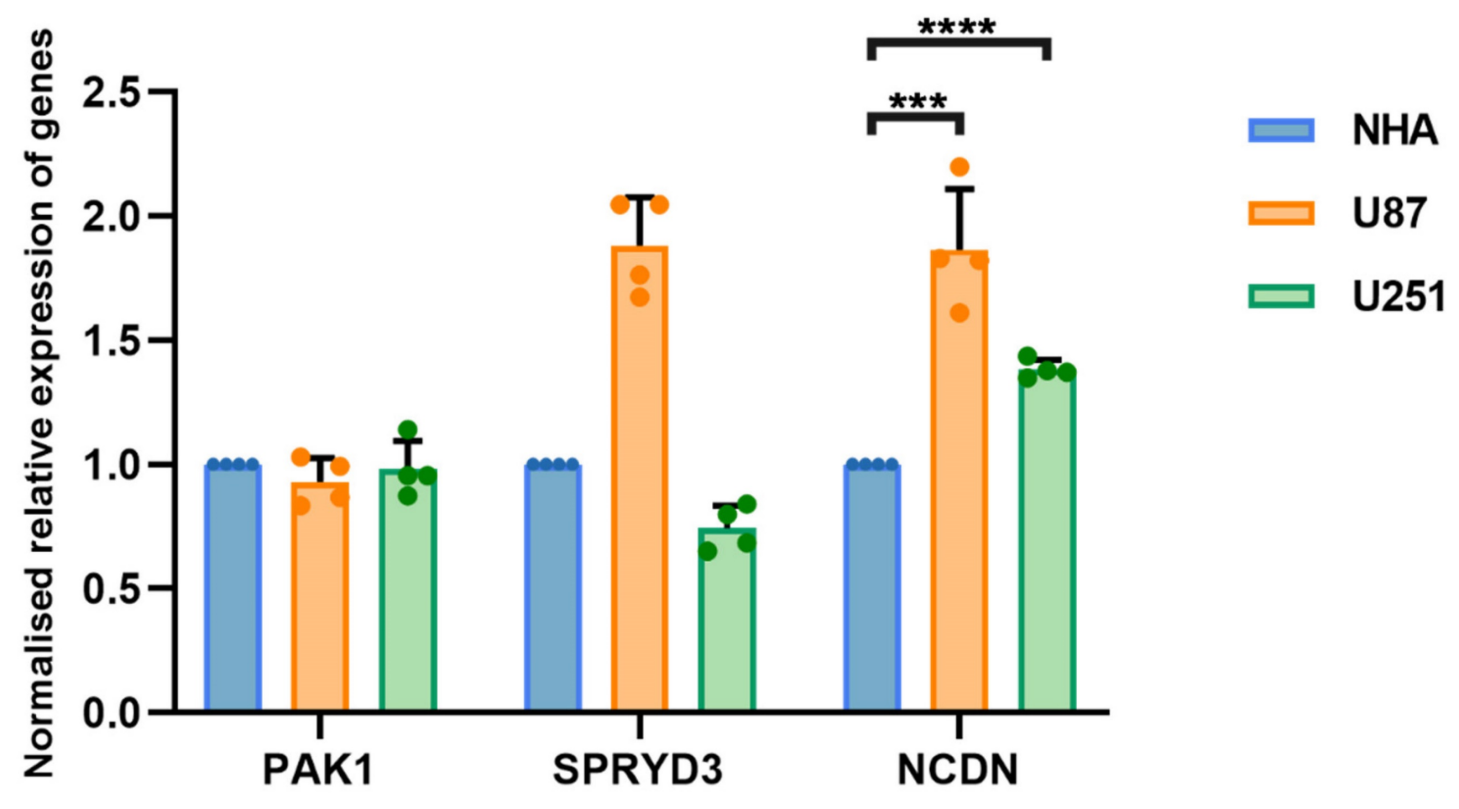
RT-qPCR analysis of *NCDN* expression in GBM cell line (U87, U251) relative to NHA cell.

The occurrence and progression of glioblastoma is attributed to the combined action of multiple genes. To clarify whether hub genes are expressed differently in glioblastoma, U87 and U251 as well as normal brain glial NHA cells were selected for experiments. Gene expression was detected by RT-qPCR. The expression of *NCDN* in U87 and U251 cells was significantly upregulated compared to NHA cells (**Figure 4**), supporting *NCDN* may have a promoting effect on glioblastoma.

### *NCDN* expression affects the migration ability of glioblastoma

**Figure 5:**
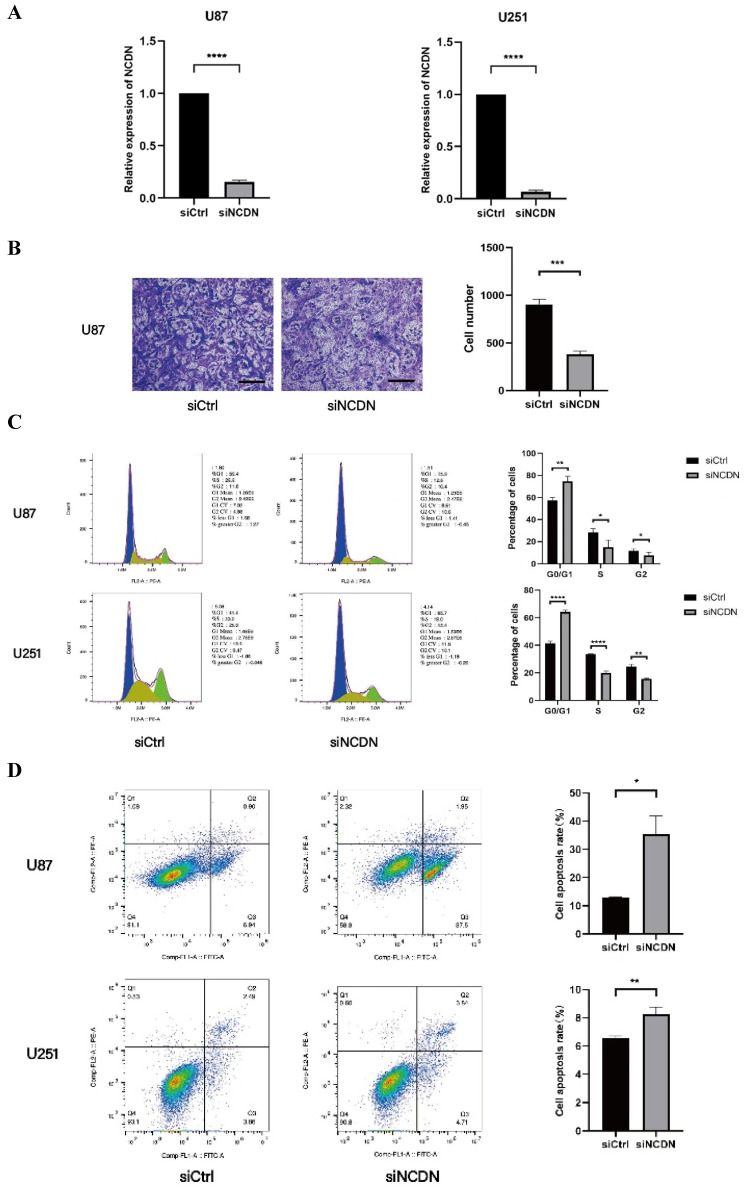
Effect of *NCDN* on GBM cell function. (A) The expression of *NCDN* in U78 and U251 transfected with *NCDN* shRNA. (B) Changes of migration ability of U87 cells after knocking down *NCDN*. Scale bar represents 100μm. The histogram counts the results of three repeated experiments. (C) Changes of cell cycle of U87 cells after knocking down* NCDN.* (D) Changes of apoptosis in U87 cells after knocking down *NCDN*.

To determine the role of *NCDN* in glioblastoma, the transwell test was performed to detect the migration capacity of U87 cells with *NCDN* knocked down (by shRNA). In the transwell assay, the mobility was lower in *NCDN* knocked-down group, clearly indicating a decreased cell migration capacity of the *NCDN* knockdown group. Our results show a promoting effect of *NCDN* has on the migratory function of glioblastoma cells (**Figure 5A, B**).

### *NCDN* is involved in the cell cycle of U251 and U87

Flow cytometry are used to determine changes in the cell cycle in U87 and U251 cell lines. Results showed that the proportion of cells in the G0/G1 stage in the *NCDN* knockdown group was significantly higher, indicating that *NCDN* knockdown inhibited the cell cycle (**Figure 5C**).

### *NCDN* has an impact on apoptosis in GBM cell lines

The effect of *NCDN* on GBM cell apoptosis was studied by flow cytometry analysis. The results showed that in both GBM cell lines, knockdown of *NCDN* induced apoptosis (**Figure 5D**).

## Discussion

Glioblastoma is an aggressive and lethal malignant brain tumor 25, 26. GBM patients who do not receive timely and effective treatment will progress to what is called a higher-grade glioma, leading to a worse prognosis 27. In this study, we identified biomarkers through bioinformatics analysis of independent patient cohort and verified them through in-vitro experiments. The results show that poor GBM prognosis is related with relatively high expression of *NCDN*. The transwell test shows that *NCDN* promoted glioma cell migration. In addition, *NCDN* knockdown promotes apoptosis and blocks cell cycle. Overall, our results illustrate that *NCDN* may be an ideal therapeutic target for inhibiting the progression of GBM.

Although the diagnosis technology, surgical intervention, and medical methods of GBM have improved in genetal, the long-term survival rate of GBM patients is still rather low, with frequent recurrence and progress 28, 29. Finding new targets may become a key challenge of novel GBM therapy. Many studies have found that various genes playing key roles in GBM and affecting a variety of cellular functions in glioblastoma 30-32. For instance, the downregulation of *circNDC80* leads to a decrease in GBM cell proliferation, migration, and invasion, which makes *circNDC80* a novel therapeutic target and prognostic biomarker for glioblastoma 33. *TRIM56* is elevated in human glioma and its product stabilizes cIAP1 protein via deubiquitination, thereby inhibiting apoptosis and promoting GBM cell proliferation 34. Upregulation of *HOTAIRM1* expression in GBM cells promotes cell migration and invasion, suggesting that targeting *HOTAIRM1* is also a possible therapeutic strategy for GBM 35. Elevated transmembrane protein TMEM230 in GBM can promote tumor cell migration, extracellular stent remodeling, and excessive blood vessels and abnormal formation of blood vessels, so *TMEM230* has the potential be a therapeutic target for inhibition of GBM tumor cells and anti-angiogenesis 36. *PDRG1* is abnormally highly expressed in GBM, promoting the migration and proliferation of GBM cells through the MEK/ERK/CD44 pathway 37.

Most current research on *NCDN* has focused on epilepsy, schizophrenia and depressive behavior, and there is limited research on oncology. One study found that the *NCDN-PDGFRA* fusion gene was present in the DNA of GBM patients, and its fusion protein could be inhibited by tyrosine kinase 38. However, no study has yet discussed the effect of *NCDN* on the malignant biological behavior of GBM. Since *NCDN* has little sequence homology with other eukaryotic proteins, little is known in terms of its function. In this study, we evaluated the effect of *NCDN* on GBM through bioinformatics analysis and cell biological function tests. We found that* NCDN* knockdown inhibits cell migration, promotes apoptosis, and induces cell cycle arrest. Our research provides a new theoretical basis for the pathogenesis and progression of GBM.

The number of normal samples in TCGA data used in this study is very small, so we will conduct additional research with balanced sample size in the future. In addition, we didn't get the expected results in the transwell experiment of U251 cells, and we will include more GBM cell lines for further research to further clarify the influence of NCDN on different GBM cell lines. Finally, we need to perform more cell and animal studies in the future.

## Conclusion

Our study shows that *NCDN* is upregulated in GBM and correlated with patient survival. In summary, *NCDN* may serve as a potential therapeutic target and biomarker for GBM treatment. Further knockdown experiments in human U251 and U87 cell lines revealed impaired cell migration, apoptosis, and cell cycle arrest. We believe that *NCDN* has the potential to become an effective target for GBM treatment. However, it is not yet clear how *NCDN* achieves this. Therefore, it is necessary to continue bioinformatics analysis and conduct more in-depth research in combination with experiments to further elucidate this.

## Figures and Tables

**Table 1:** The sample size and clinical information for GBM dataset.

Characteristics	Deceased (*n*=286)		Living (*n*=79)	Total (*n*=365)	*P*
**Age, years**					
Mean±SD		62.00±13.56	56.70±15.36	60.85±14.12	
Median[min-max]		63.00[14.00,89.00]	59.00[21.00,83.00]	62.00[14.00,89.00]	
**Gender**					0.51
		
Female		109(29.86%)	34(9.32%)	143(39.18%)	
Male		177(48.49%)	45(12.33%)	222(60.82%)	
**Type**					0.66
		
Primary Tumor		283(77.53%)	79(21.64%)	362(99.18%)	
Recurrent Tumor		2(0.55%)	0(0.0e+0%)	2(0.55%)	
Solid Tissue Norma		1(0.27%)	0(0.0e+0%)	1(0.27%)	
					

Values are n (%), unless otherwise noted.

## Abbreviations

GBM: Glioblastoma
TCGA: The Cancer Genome Atlas
GO: Gene Ontology
WGCNA: Weighted Gene Co-expression Network Analysis
MM: Module Members
GS: Gene Significance
DEGs: Differentially Expressed Genes
